# Correction Method for Line Extraction in Vision Measurement

**DOI:** 10.1371/journal.pone.0127068

**Published:** 2015-05-18

**Authors:** Mingwei Shao, Zhenzhong Wei, Mengjie Hu, Guangjun Zhang

**Affiliations:** Beihang University, Key Laboratory of Precision Opto-mechatronics Technology, Ministry of Education, Beijing, 100191, China; Xiamen University, CHINA

## Abstract

Over-exposure and perspective distortion are two of the main factors underlying inaccurate feature extraction. First, based on Steger’s method, we propose a method for correcting curvilinear structures (lines) extracted from over-exposed images. A new line model based on the Gaussian line profile is developed, and its description in the scale space is provided. The line position is analytically determined by the zero crossing of its first-order derivative, and the bias due to convolution with the normal Gaussian kernel function is eliminated on the basis of the related description. The model considers over-exposure features and is capable of detecting the line position in an over-exposed image. Simulations and experiments show that the proposed method is not significantly affected by the exposure level and is suitable for correcting lines extracted from an over-exposed image. In our experiments, the corrected result is found to be more precise than the uncorrected result by around 45.5%. Second, we analyze perspective distortion, which is inevitable during line extraction owing to the projective camera model. The perspective distortion can be rectified on the basis of the bias introduced as a function of related parameters. The properties of the proposed model and its application to vision measurement are discussed. In practice, the proposed model can be adopted to correct line extraction according to specific requirements by employing suitable parameters.

## Introduction

In the field of remote sensing, curvilinear structures (lines for short) are extracted from aerial and satellite images to determine key information such as roads and rivers [1–4]. Further, in the field of medical image analysis, line extraction facilitates the detection of blood vessels and nerves, and the obtained information is important for medical diagnosis [5–9]. Moreover, in some fields of vision measurement, including 3D reconstruction using structured light, stereo reconstruction [10–15], 3D Object Retrieval and Recognition [16–22], and so on, the image of the feature that reflects information on the target morphology is often a line. Therefore, line extraction is an indispensable technique in various fields.

Thus far, various methods for line extraction have been proposed. Lines can be extracted using skeletons, whereby the candidate skeleton is extracted from the Euclidean distance map [23]. The skeletal points of the candidate skeleton are classified into three types: ridge, ravine, and stair. Based on the classification, the line regions are reconstructed. In [24], the clustering of principal curves was adopted to detect curvilinear features in spatial point patterns. Based on the theory of hierarchical, agglomerative, and related iterative relocation, line features in spatial point patterns can be determined in a straightforward manner. A line extraction method used primarily for detecting roads in spaceborne synthetic aperture radar images is presented in [25]. This method is based on a genetic algorithm, and it can detect roads accurately via curve segment extraction and postprocessing. Thus, the three different methods described above employ three distinct theories. However, problems such as extensive computation and significant bias persist in line extraction. Owing to the large number of classified candidate points, optimization is necessary in some methods; thus, the extraction may be time-consuming. Moreover, the extraction may involve additional noise when a line is asymmetrical. Therefore, models for asymmetric bar-shaped [26], parabolic, and Gaussian line profiles have been proposed to describe the profile of a line in [27] (Steger’s method). The center of the profile can be determined by the zero crossing of its first-order derivative, whereas the edge can be determined by the zero crossing of its second-order derivative. Partial derivatives of a real image are computed by convolving the image with the corresponding partial derivative of a normal Gaussian kernel. A bias function that can be obtained by the bisection method [28] is used to rectify the center and edge of the line. In addition, the bar-shaped model has been used to extract line positions of light stripes [29]. Owing to its high accuracy, this method is widely used for image processing in the field of vision measurement.

In vision measurement systems based on line-structured light, over-exposure is ubiquitous owing to intense illumination and extensive reflection. Because of the finite sensitivity of the vision sensor, it becomes saturated easily; thus, over-exposure of images is inevitable. In [30], a license-plate detection method has been proposed to facilitate the detection of license plates from over-exposed images. This method involves the following steps: converting a color image into a grayscale image, equalizing the image, detecting the edges, checking the black pixel ratio, verifying the license plate, and outputting the license plate. In [31], an approach for correcting over-exposure in photographs has been introduced; it is based on the separate recovery of color and lightness. However, these methods are effective only in some specific fields because they are not based on a special model, as defined in [27]. But in Steger’s method, the over-exposure is not considered. The center of the line profile is normally with additional error in the over-exposure image.

Moreover, most cameras used for capturing images are based on a projective model; therefore, perspective distortion of the captured image is inevitable, and it results in inaccurate line extraction. The typical projective model is shown in Fig 1. The centerline of a curve in the scene is *L*, whereas the centerline of its image is *l*. The line *l’* in the image is the projection of *L* on the image plane. Further, *o*-*xy* is the image coordinate system, whereas *O*-*XYZ* is the camera coordinate system. Owing to the existence of perspective distortion, the lines *l* and *l’* are not coincident. Similarly, a circle in the scene also suffers from the same problem. In [32], the perspective distortion of a circular center in the image plane has been modeled. In [33], the perspective distortion of an elliptical center, which is a more general case, has been analyzed on the basis of projective transformation and analytic geometry. Thus, perspective distortion is a significant issue that needs to be rectified from the viewpoint of line extraction, especially in some industrial measurement fields that require high precision.

**Fig 1:**
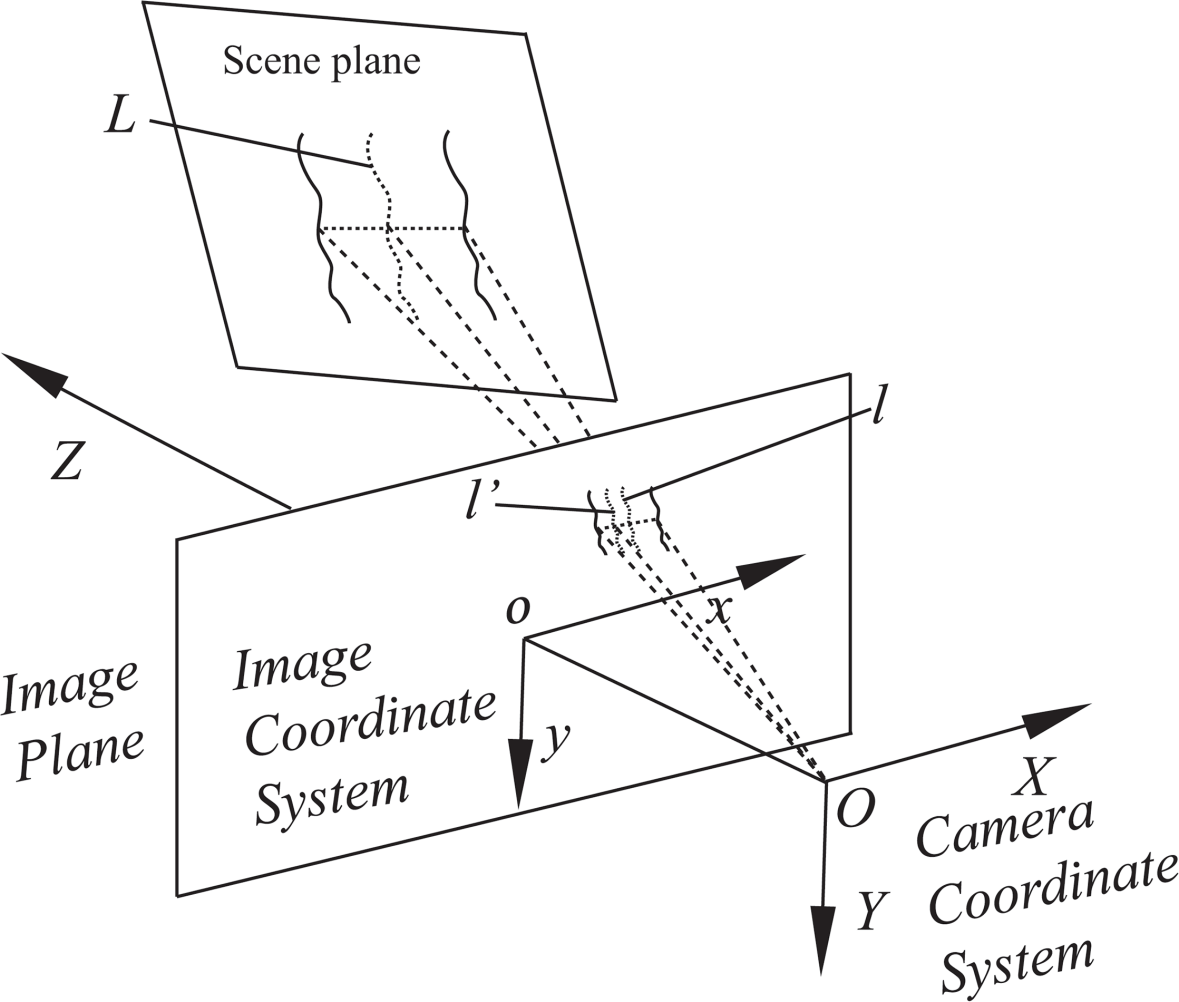
Typical projective model of a camera.

In this paper, we propose a new model for correcting line positions in an over-exposed image. The proposed model is based on the Gaussian model of Steger’s method, with the additional capability of suitably fitting line profiles in over-exposed images. As a result of this correction procedure, the center of the line profile becomes close to the ideal/actual center (as shown in Fig 2). We also discuss the related properties of perspective distortion. In addition, we describe the relationship between the centerline of a scene and the centerline of its image, which can serve as a reference for correcting the bias introduced by perspective distortion. Further, we discuss the practical applications of the proposed method, including its application to vision measurement. Finally, we describe some simulations and experiments conducted to verify the validity of our method. Because the line position is critical in vision measurement, obtaining the line position is the primary objective of this study.

**Fig 2:**
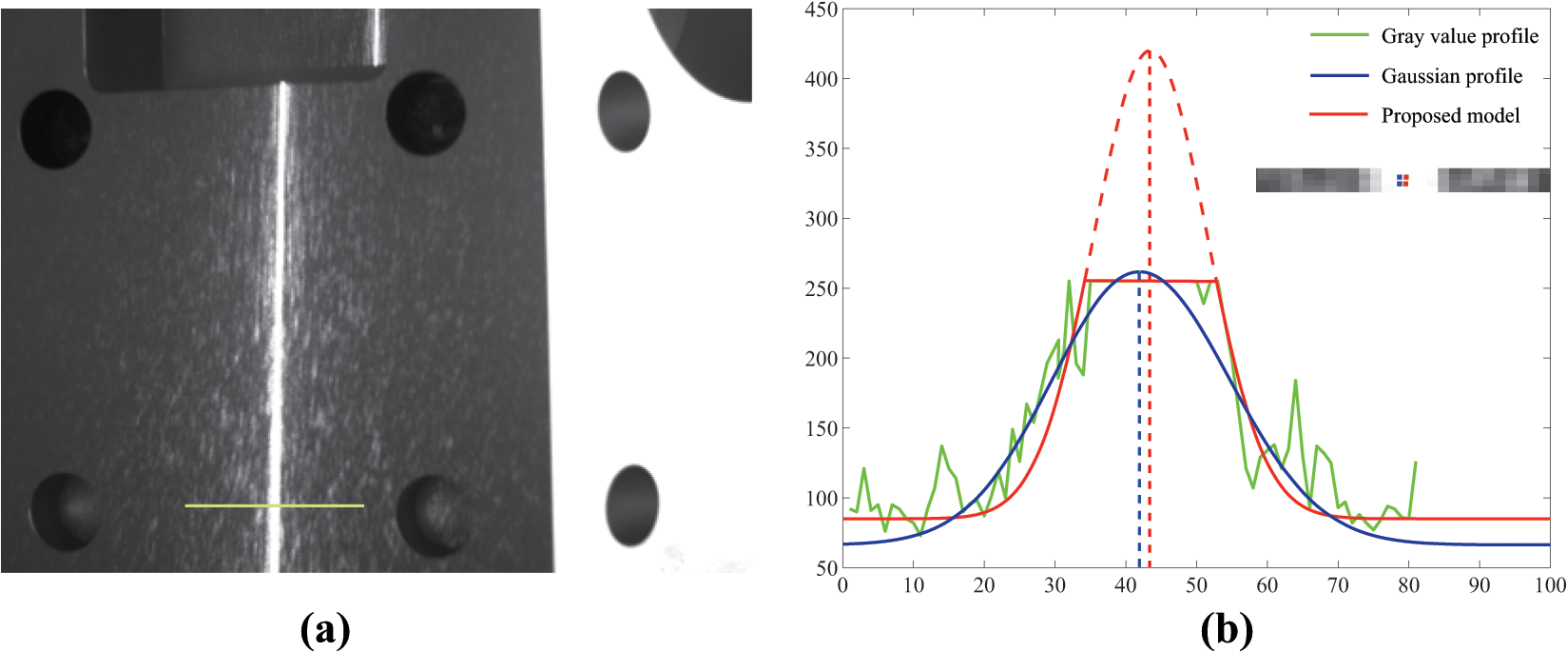
(a) Over-exposure image, (b) extraction result in over-exposure image.

## Correction of Line Position in Over-Exposed Image

### A) Model description

#### a) Steger’s line extraction model

As lines exhibit a characteristic profile across the line at each point, the problems concerning the extraction of lines are essentially one-dimensional in nature. The related analysis can be carried out for one-dimensional line profile. When considering one-dimensional line profile, a bar-sharped line model, a parabolic line model and a Gaussian line model are used to fit the profile and remove the bias in Steger’s method respectively[26,27]. The images of the three models in Steger’s method are shown in Fig 3.

**Fig 3:**
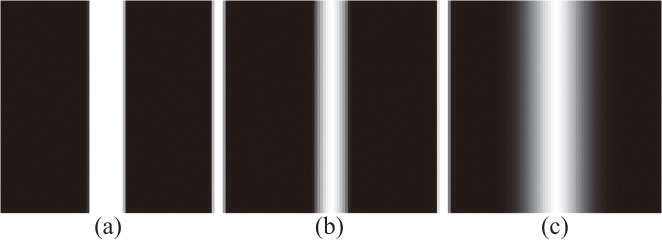
(a) Image of a bar-shaped, (b) image of a parabolic, and (c) image of a Gaussian line with equal line widths.

The functions of the three models stated in Steger’s method are listed below: *f*_b_(*x*) denotes the bar-shaped line profile, *f*_p_(*x*) denotes the parabolic line profile, and *f*_g_(*x*) denotes the Gaussian line profile.

$$f_{b}(x)=\{\begin{array}{c} 0,(-\infty ,-w)\\ 1,[-w,+w]\\ a,(+w,+\infty ) \end{array},$$ (1)

$$f_{p}(x)=\{\begin{array}{l} 0,(-\infty ,-w)\\ 1-{\left(\frac{x}{w}\right)}^{2},[-w,0]\\ 1-(1-a){\left(\frac{x}{w}\right)}^{2},[0,+w]\\ a,(+w,+\infty ) \end{array},$$ (2)

$$f_{g}(x)=\{\begin{array}{l} e^{-\frac{x^{2}}{2w^{2}}},(-\infty ,0]\\ a+(1-a)e^{-\frac{x^{2}}{2w^{2}}},(0,+\infty ) \end{array}$$ (3)

where *w* denotes the width of the profile and *a* (0 ≤ *a* <1) denotes the asymmetry of the profile. Among these three models, the Gaussian line profile is the most precise model for line extraction after correction, whereas the bar-shaped line profile is simplest one.

To extract the line position in an over-exposed image, the bar-shaped profile and the Gaussian profile are adopted to fit the profile, including the ideal and real profiles. The related fitting results are shown in Fig 4.

**Fig 4:**
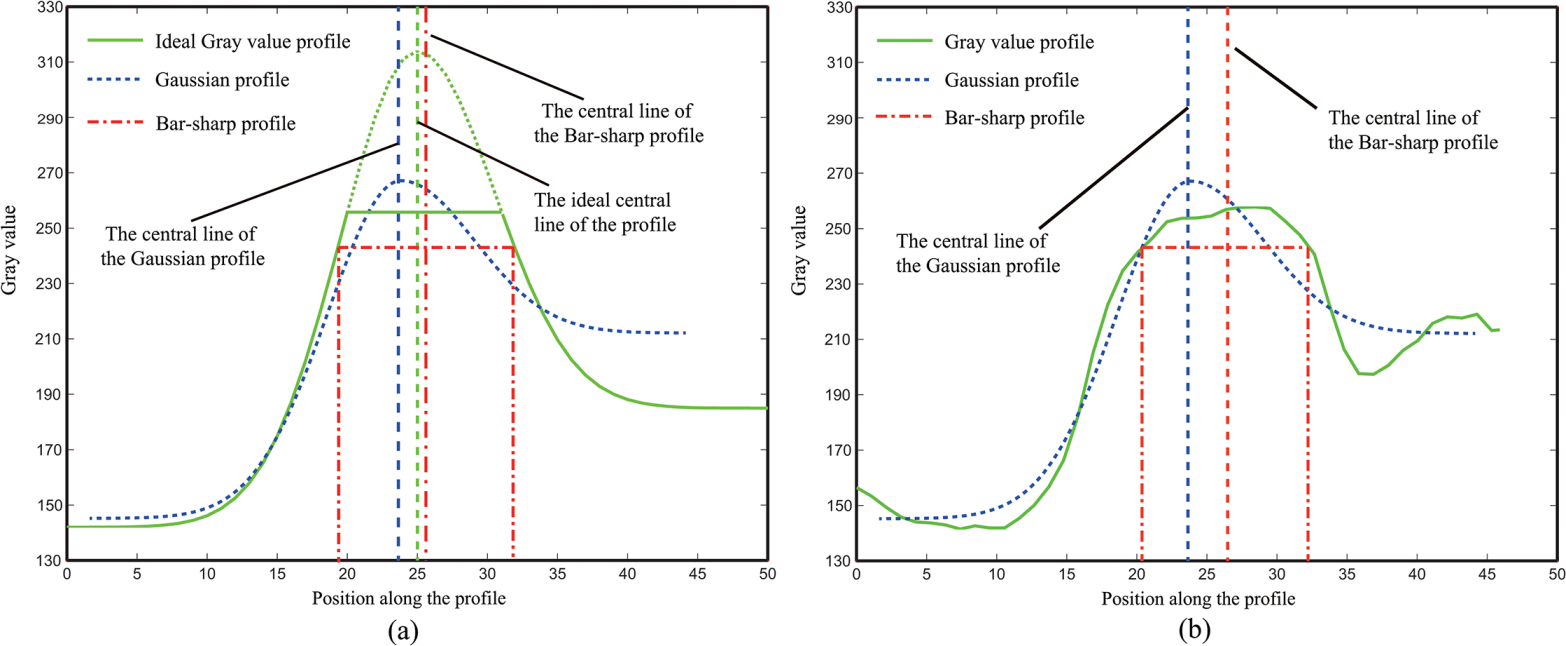
(a) Ideal over-exposure model and fitting result using the bar-shaped profile and the Gaussian profile, (b) line profile in the over-exposed image.

In Fig 4, there is a certain bias between the ideal center and the fitted one when either the bar-shaped line profile or the Gaussian line profile is used. Image saturation is the main reason for this phenomenon, as shown in Fig 4(A). Similar problems also exist in real images, as shown in Fig 4(B). Thus, the models stated in Steger’s method cannot fit the line profile in an over-exposed image properly. Therefore, a bias is introduced between the extracted center and the ideal/actual one.

#### b) Over-exposure model

Owing to the saturation of over-exposed images, Steger’s line extraction models, including the bar-shaped, parabolic, and Gaussian line profiles, do not allow accurate fitting of the line profile. Therefore, we propose a new model, namely, the over-exposure model, based on Steger’s Gaussian line profile.

As shown in Fig 5, let us assume that *T* is the saturation value in the over-exposure model. From Eq (3), *f*(*x*) is given by

$$f(x)=\{\begin{array}{l} e^{-\frac{x^{2}}{2w^{2}}},(-\infty ,-\sqrt{-2w^{2}\;\text{ln}\;T})\\ T,(-\sqrt{-2w^{2}\;\text{ln}\;T},\sqrt{-2w^{2}\;\text{ln}\frac{T-a}{1-a}})\\ a+(1-a)e^{-\frac{x^{2}}{2w^{2}}},(\sqrt{-2w^{2}\;\text{ln}\frac{T-a}{1-a}},+\infty ) \end{array},$$ (4)

where *a* and *w* are defined as in Eq (3). The relation between *T* and *a* is 1 > *T* > *a* > 0 in the scale space. The description of the real profile requires a scale factor related to the description in the scale space. The fitness of a profile in the real image using the proposed method is plotted in Fig 5.

**Fig 5:**
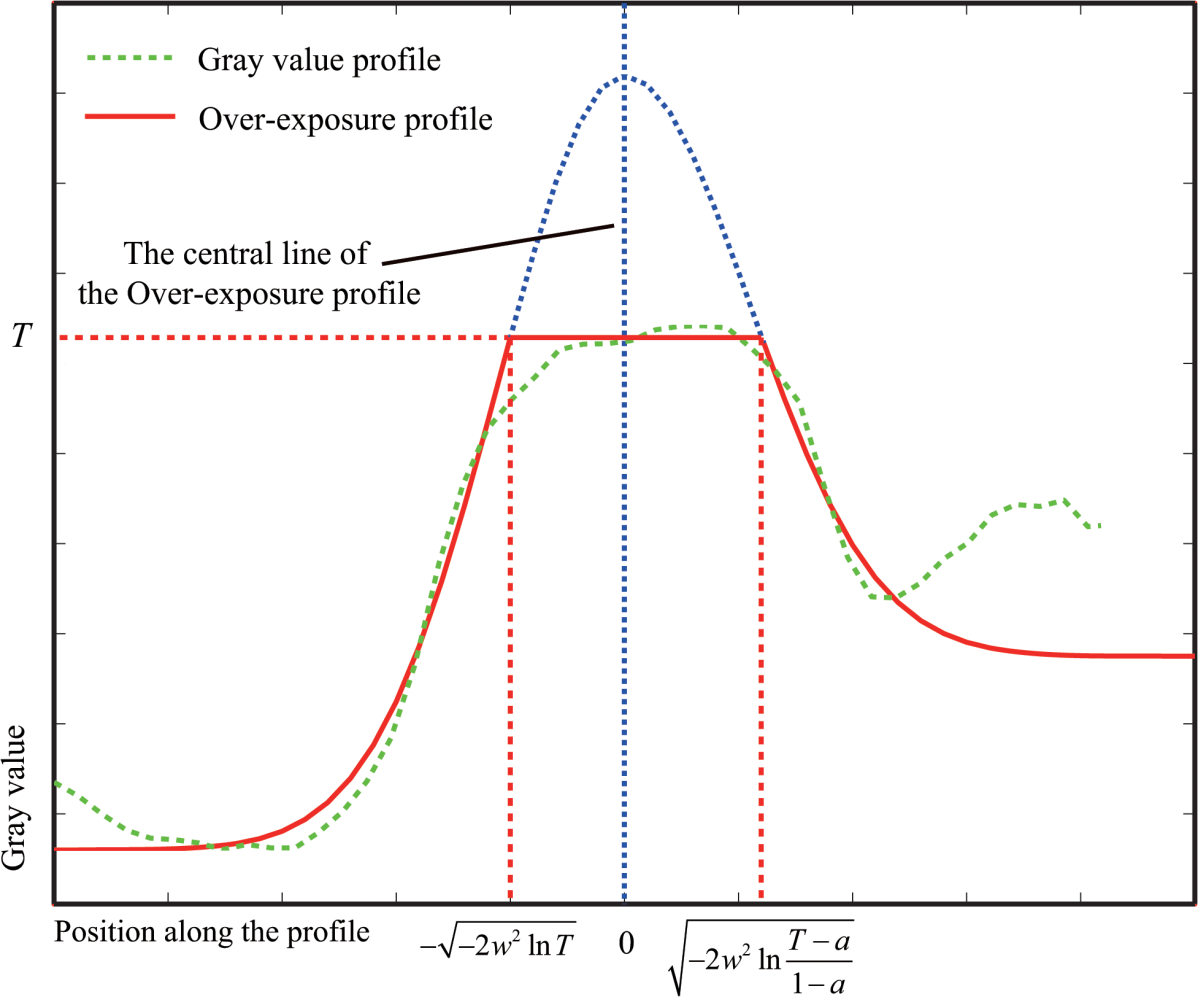
Fitness of a real profile using the proposed model.

The center of the gray value profile is determined by the zero crossing of the first-order derivative (*f’*(*x*) = 0), i.e., the local maximum (for bright lines) or local minimum (for dark lines) of the gray value profile. For real images, this criterion must be augmented with a criterion for selecting salient lines representing the noise involved. This can be achieved with a threshold on |*f”*(*x*)|, as mentioned in Steger’s method, i.e., by requiring that *f”*(*x*) << 0 for bright lines and *f”*(*x*) >> 0 for dark lines. Furthermore, derivatives of the real image are estimated by convolving the image with the derivative of a normal Gaussian kernel [34]. The normal Gaussian kernel and its derivatives are given by

$$g(x,\sigma )=\frac{1}{\sqrt{2\pi }\sigma }e^{-\frac{x^{2}}{2\sigma^{2}}},$$ (5)

$$g'(x,\sigma )=\frac{-x}{\sqrt{2\pi }\sigma^{3}}e^{-\frac{x^{2}}{2\sigma^{2}}},$$ (6)

$$g''(x,\sigma )=\frac{x^{2}-\sigma^{2}}{\sqrt{2\pi }\sigma^{5}}e^{-\frac{x^{2}}{2\sigma^{2}}}.$$ (7)

In the scale space, the description of the profile can be determined by convolving the gray value profile with the corresponding derivative of the normal Gaussian kernel. The scale space description is given by

$$\begin{array}{l} r(x,\sigma ,w,a,T)=f(x)*g(x,\sigma )\\ =\sqrt{2\pi }wg(x,\sqrt{w^{2}+\sigma^{2}})+[1-a\sqrt{2\pi }wg(x,\sqrt{w^{2}+\sigma^{2}})]\phi (-x_{2}+\frac{xw^{2}}{w^{2}+\sigma^{2}},\frac{w\sigma }{\sqrt{w^{2}+\sigma^{2}}})\\ -\phi (x_{1}+\frac{xw^{2}}{w^{2}+\sigma^{2}},\frac{w\sigma }{\sqrt{w^{2}+\sigma^{2}}})+(a-T)\phi (x-x_{2})+T\phi (x+x_{1}) \end{array},$$ (8)

$$\begin{array}{l} r'(x,\sigma ,w,a,T)=f(x)*g'(x,\sigma )\\ =\sqrt{2\pi }wg'(x,\sqrt{w^{2}+\sigma^{2}})*[1-a\phi (-x_{2}+\frac{xw^{2}}{w^{2}+\sigma^{2}},\frac{w\sigma }{\sqrt{w^{2}+\sigma^{2}}})]\\ +g(-x_{2}+\frac{xw^{2}}{w^{2}+\sigma^{2}},\frac{w\sigma }{\sqrt{w^{2}+\sigma^{2}}})[1-a\sqrt{2\pi }wg(x,\sqrt{w^{2}+\sigma^{2}})]\\ -\frac{w^{2}}{w^{2}+\sigma^{2}}g(x_{1}+\frac{xw^{2}}{w^{2}+\sigma^{2}},\frac{w\sigma }{\sqrt{w^{2}+\sigma^{2}}})+(a-T)g(x-x_{2})+Tg(x+x_{1}) \end{array},$$ (9)

$$\begin{array}{l} r''(x,\sigma ,w,a,T)=f(x)*g''(x,\sigma )\\ =\sqrt{2\pi }wg''(x,\sqrt{w^{2}+\sigma^{2}})*[1-a\phi (-x_{2}+\frac{xw^{2}}{w^{2}+\sigma^{2}},\frac{w\sigma }{\sqrt{w^{2}+\sigma^{2}}})]\\ -{\left(\frac{w^{2}}{w^{2}+\sigma^{2}}\right)}^{2}g'(-x_{2}+\frac{xw^{2}}{w^{2}+\sigma^{2}},\frac{w\sigma }{\sqrt{w^{2}+\sigma^{2}}})[1-a\sqrt{2\pi }wg(x,\sqrt{w^{2}+\sigma^{2}})]\\ -2a\sqrt{2\pi }wg'(x,\sqrt{w^{2}+\sigma^{2}})g(-x_{2}+\frac{xw^{2}}{w^{2}+\sigma^{2}},\frac{w\sigma }{\sqrt{w^{2}+\sigma^{2}}})\frac{w^{2}}{w^{2}+\sigma^{2}}\\ -{\left(\frac{w^{2}}{w^{2}+\sigma^{2}}\right)}^{2}g'(x_{1}+\frac{xw^{2}}{w^{2}+\sigma^{2}},\frac{w\sigma }{\sqrt{w^{2}+\sigma^{2}}})+(a-T)g'(x-x_{2})+Tg'(x+x_{1}). \end{array},$$ (10)

The related notations are defined as

$$x_{1}=-\sqrt{-2w^{2}\;\text{ln}\;T},x_{2}=\sqrt{-2w^{2}\;\text{ln}\frac{T-a}{1-a}},\phi (x,\sigma )=\int_{-\infty }^{x}e^{-\frac{t^{2}}{2\sigma^{2}}}dt.$$

### B) Removal of bias

As the line position is the necessary information in vision measurement, we analyze the bias of the center of the profile in an over-exposed image as well as the effect of the saturation value *T*. The center of the profile is determined from the zero crossing of the first-order derivative, i.e., *r*'(*x*,*σ*,*w*,*a*,*T*) = 0. Because direct determination is difficult, the centerline is computed using a numerical root finding algorithm, as described in Steger’s method. We use the bisection method [28] in the proposed extraction method. The following proposition is obtained from [27]: *if both σ and w are scaled by the same constant factor s*, *the line and edge locations will be sl*, *se*_*l*_, *and se*_*r*_, where *l* is the line position, *e*_l_ is the left edge point, and *e*_r_ is the right edge point. Thus, bias analysis can be performed for *σ* = 1, and all other values can be obtained via simple multiplication by the actual scale *σ*.

#### a) Effect of *T* on bias

In this section, we analyze the effect of the parameter *T* on the bias, under the conditions of asymmetry and symmetry. The relationship between the convolution of the profile with the Gaussian kernel function and the parameter *T* is shown in Fig 6.

**Fig 6:**
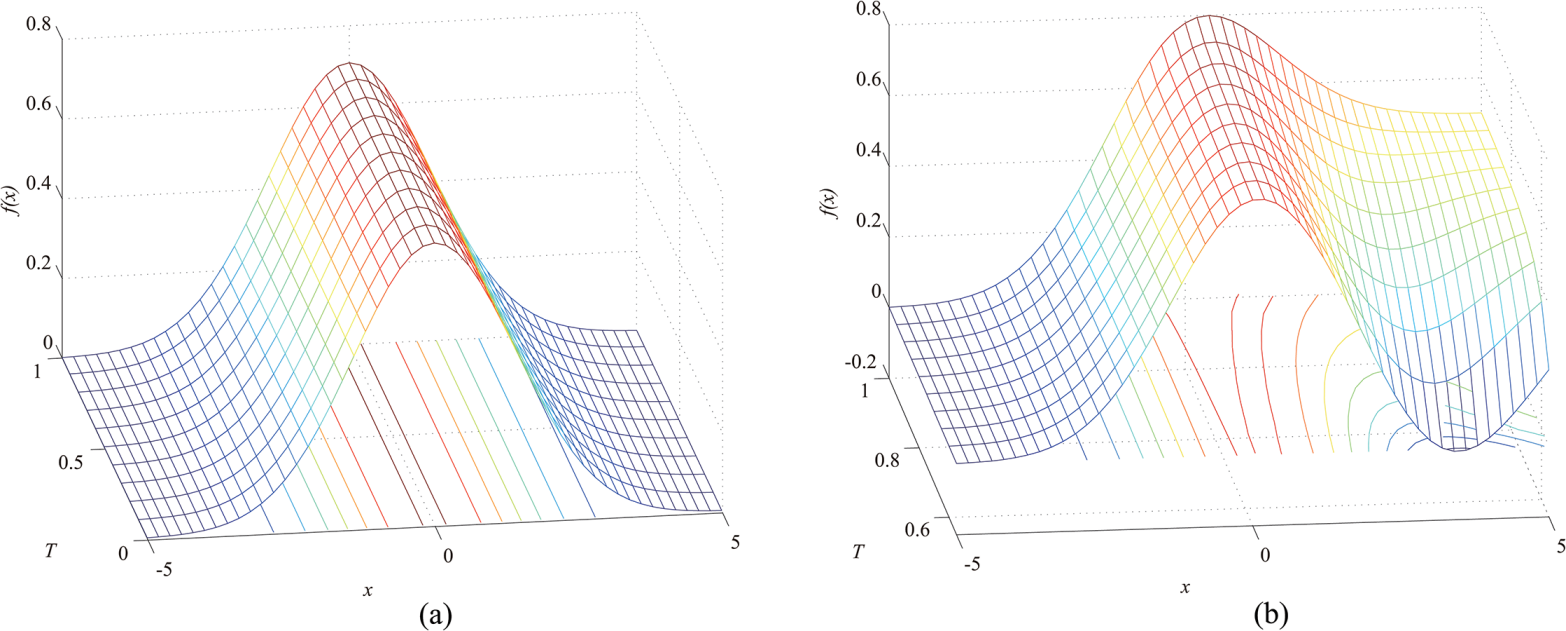
(a) Gray value as a function of *T* and position *x* in the case of symmetry; (b) Gray value as a function of *T* and position *x* in the case of asymmetry.

The line position, i.e., the maximum of the profile, is invariant in the case of symmetry. In contrast, in the asymmetric condition, the maximum of the profile varies according to *T*. Thus, in the case of asymmetry, correction should be performed in order to obtain a precise position.

#### b) Correction in asymmetric condition

The profile in the over-exposure model is a part of the Gaussian line profile. Let the entire Gaussian line profile be *R*_g_(*x*,*σ*,*w*,*a*) and the missing part be $R_{g}^{d}(x,\sigma ,w,a,T)$. Then, the expression for *r* can be rewritten as

$$r(x,\sigma ,w,a,T)=f(x,\sigma ,w,a,T)*g(x,\sigma )=(R_{\text{g}}(x,\sigma ,w,a)-R_{g}^{d}(x,\sigma ,w,a,T))*g(x,\sigma ).$$ (11)

When *T* << 1, the extreme point of *r* is not unique, i.e., the center of the profile will be incorrect. Then, $R_{g}^{d}(x,\sigma ,w,a,T)$ can be substituted for *f*(*x*,*σ*,*w*,*a*,*T*), because they have the same center point. Further, the description of $R_{g}^{d}(x,\sigma ,w,a,T)$ can be determined in a similar manner as the description of *f*(*x*,*σ*,*w*,*a*,*T*):

$$\begin{array}{l} r_{g}^{d}(x,\sigma ,w,a,T)=-R_{g}^{d}(x,\sigma ,w,a,T)*g(x,\sigma )\\ =\phi (-x_{2}+\frac{xw^{2}}{w^{2}+\sigma^{2}},\frac{w\sigma }{\sqrt{w^{2}+\sigma^{2}}})-\phi (x_{1}+\frac{xw^{2}}{w^{2}+\sigma^{2}},\frac{w\sigma }{\sqrt{w^{2}+\sigma^{2}}})-T\phi (x-x_{2})+T\phi (x+x_{1}) \end{array},$$ (12)

$$\begin{array}{l} r_{g}^{d'}(x,\sigma ,w,a,T)=-R_{g}^{d}(x,\sigma ,w,a,T)*g'(x,\sigma )\\ =g(-x_{2}+\frac{xw^{2}}{w^{2}+\sigma^{2}},\frac{w\sigma }{\sqrt{w^{2}+\sigma^{2}}})-\frac{w^{2}}{w^{2}+\sigma^{2}}g(x_{1}+\frac{xw^{2}}{w^{2}+\sigma^{2}},\frac{w\sigma }{\sqrt{w^{2}+\sigma^{2}}})\\ -Tg(x-x_{2})+Tg(x+x_{1}) \end{array},$$ (13)

$$\begin{array}{l} r_{g}^{d''}(x,\sigma ,w,a,T)=-R_{g}^{d}(x,\sigma ,w,a,T)*g''(x,\sigma )\\ =-{\left(\frac{w^{2}}{w^{2}+\sigma^{2}}\right)}^{2}g'(-x_{2}+\frac{xw^{2}}{w^{2}+\sigma^{2}},\frac{w\sigma }{\sqrt{w^{2}+\sigma^{2}}})\\ -{\left(\frac{w^{2}}{w^{2}+\sigma^{2}}\right)}^{2}g'(x_{1}+\frac{xw^{2}}{w^{2}+\sigma^{2}},\frac{w\sigma }{\sqrt{w^{2}+\sigma^{2}}})-Tg'(x-x_{2})+Tg'(x+x_{1}). \end{array},$$ (14)

The related notations are defined as in Eq (10).

It is known that the edge points of the line are the zero crossings of its second-order derivate, while the line positions are the zero crossings of its first-order derivate. In order to eliminate the scale effect, the symbol *λ* is introduced:

$$\lambda =\left|r_{g}^{d'}(e_{l},\sigma ,w,a,T)\right|/\left|r_{g}^{d'}(e_{r},\sigma ,w,a,T)\right|.$$ (15)

The bias function is a map from *v*_*σ*_ and *λ* to *w*_*σ*_, *T*, and *a*, where *v*_*σ*_ is the width of the profile, i.e., *v*_*σ*_ = |*e*_*l*_ − *e*_*r*_|; thus, the correction can be determined [27].

Since a function from *R* ^2^ to *R* ^3^ can only be injective, it is difficult to analyze the bias removal function. For this reason, the parameter *w* is fixed, and in this case, the bias removal function is from *R* ^2^ to *R* ^2^, which is surjective. In our paper, we analyze only the relationships of *v*_*σ*_, *λ*, and the bias with the parameters *T* and *a* when *w* equals 1, 2, and 3, respectively. As in the case of the approach described in [27], the results for other values can be obtained by interpolation. The relations are shown in Figs 7, 8 and 9. Our profile involves the constraint 1 > *T* > *a* > 0, but the data are expanded by interpolation in these illustrations for the sake of clarity.

**Fig 7:**
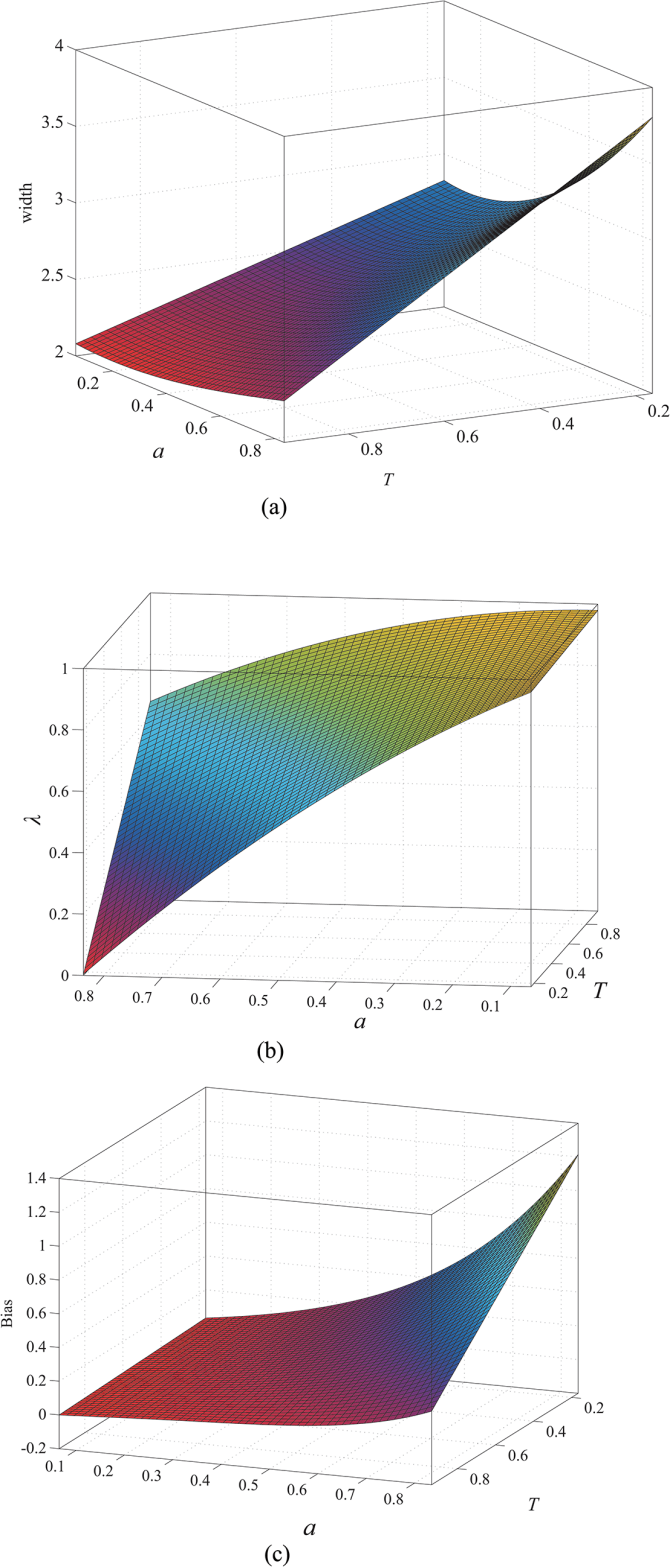
When *w* = 1, (a) width as a function of *a* and *T*; (b) *λ* as a function of *a* and *T*; (c) bias as a function *a* and *T*.

**Fig 8:**
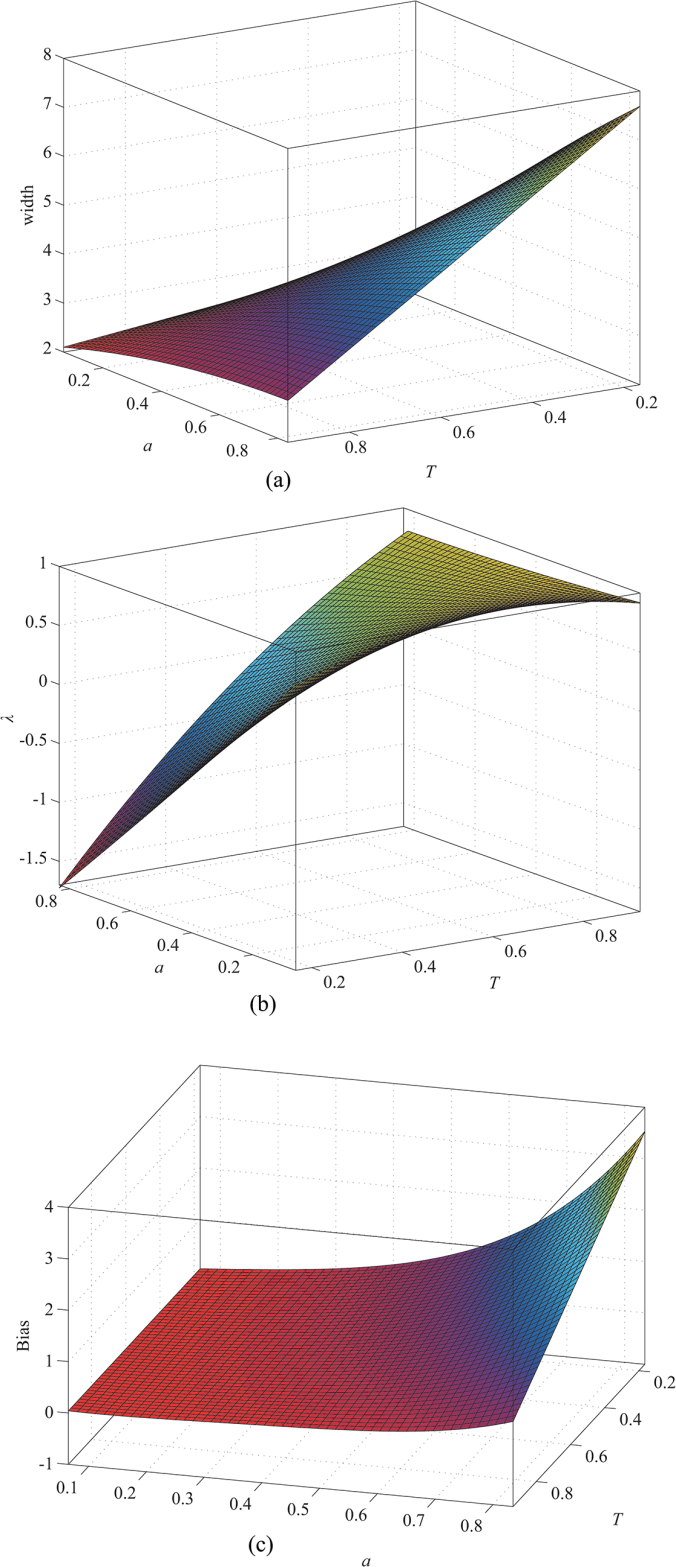
When *w* = 2, (a) width as a function of *a* and *T*; (b) *λ* as a function of *a* and *T*; (c) bias as a function of *a* and *T*.

**Fig 9:**
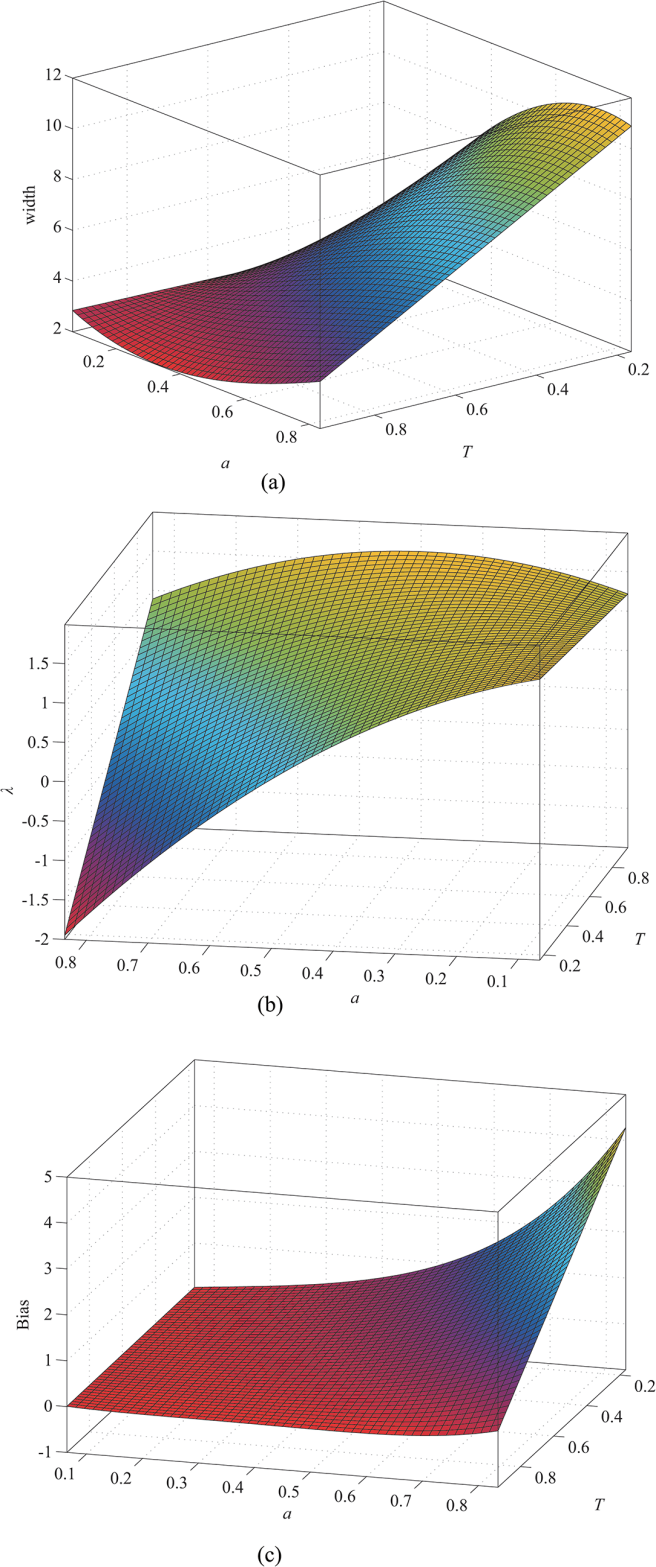
When *w* = 3, (a) width as a function of *a* and *T*; (b) *λ* as a function of *a* and *T*; (c) bias as a function of *a* and *T*.

The results indicate that the line width increases as *a* increases or *T* decreases. Thus, the line width increases from Fig 7(A) to Fig 9(A). Therefore, the line width is proportional to *w*. From Fig 7(B), *λ* increases as *a* decreases or *T* increases. Similarly, we can conclude that *λ* is proportional to *w* from Figs 7(B), 8(B) and 9(B). The bias, which is shown in Figs 7(C), 8(C) and 9(C) when *w* equals 1, 2, and 3, respectively, is proportional to *a* and *w* but inversely proportional to *T*. Therefore, correction is necessary for accurate extraction of the line position, especially when *a* and *w* are too large or *T* is too small.

## Correction of Perspective Distortion

### A) Related properties

As the camera model is projective, perspective distortion exists in the captured line image, i.e., the line position of the image does not correspond with the projection of the line position of the scene on the image plane. The perspective distortion in the camera model is shown in Fig 10.

**Fig 10:**
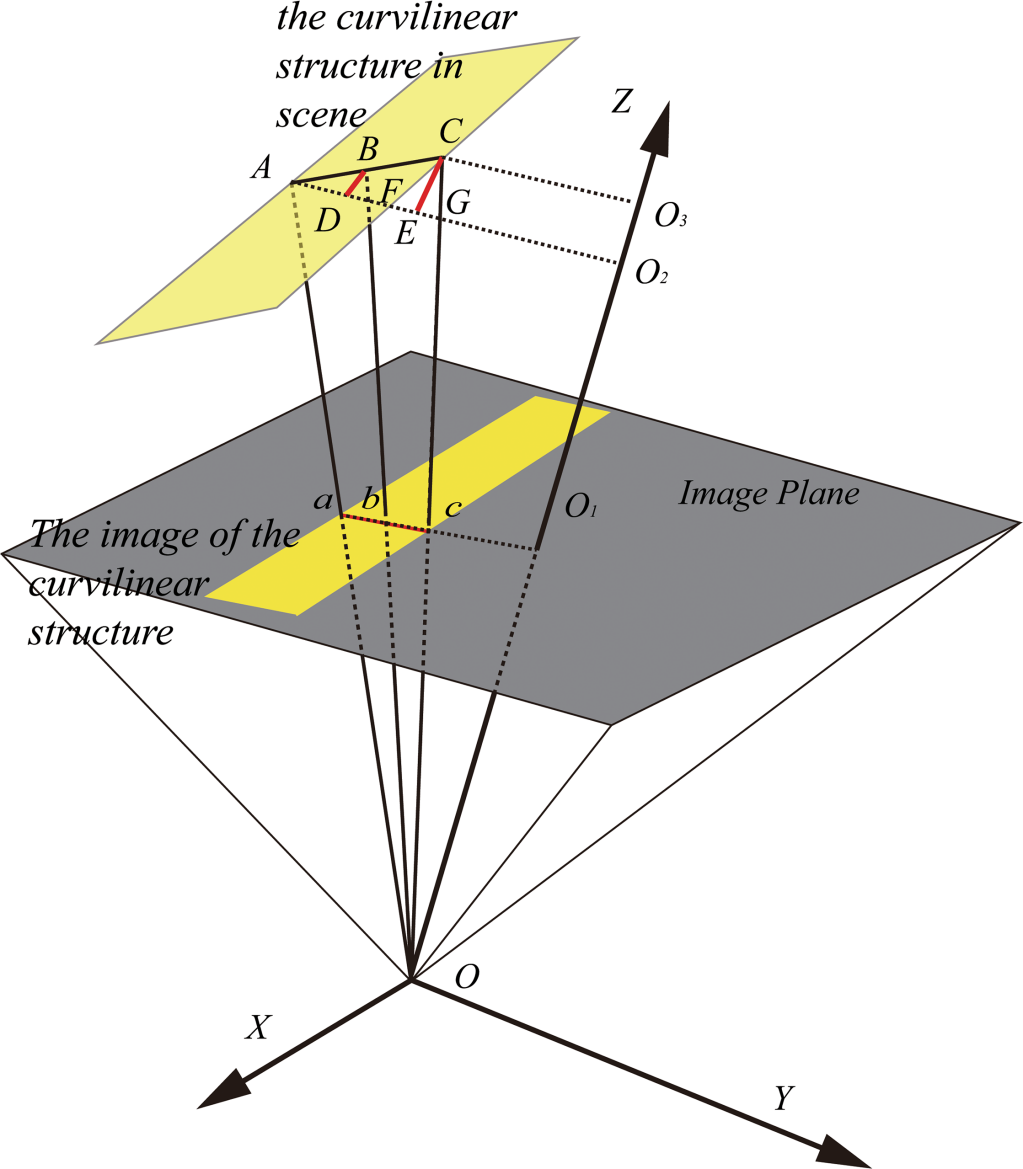
Perspective distortion in the camera model.

In Fig 10, *O* is the optical center of the camera and *O*-*XYZ* is the camera coordinate system (*CCS*). The profile of the line is denoted by line segment *AC*, and its midpoint is point *B*. The points corresponding to points *A*, *B*, and *C* on the image plane are points *a*, *b*, and *c*, respectively. Two lines parallel to *ac* and passing separately through point *A* and point *C* intersect with line *OO*_1_ at point *O*_2_ and point *O*_3_, respectively. Point *F* and point *G* are the intersections of line *AO*_2_ with line *OB* and line *OC*, separately. The vertical lines that pass through point *B* and point *C* intersect with the line *AO*_2_ at point *D* and point *E*, respectively. Owing to the limited field of view (*FOV*) of the lens, the range of angle *BAD* (0~90°) and angle *GOO*_1_ (0~45°) can be determined; we define angle *BAD* as *θ*, angle *GOO*_1_ as *β*, the width of the profile as *l*, and the distance from the line to the image plane as *d*. The following relation is obtained:

$$\Delta DBF≃\Delta O_{2}OF.$$ (16)

Then,

$$\frac{DB}{OO_{2}}=\frac{DF}{O_{2}F}\Rightarrow \frac{l\;\text{sin}\;\theta /2}{f+d}=\frac{l\;\text{cos}\;\theta /2+\left|EF\right|}{(f+d+l\;\text{sin}\;\theta )\;\text{tan}\;\beta -\left|EF\right|}.$$ (17)

Therefore, the length of line segment *EF* is

$$\left|EF\right|=\frac{l\;\text{sin}\;\theta \;\text{tan}\;\beta (f+d+l\;\text{sin}\;\theta )-l\;\text{cos}\;\theta (f+d)}{l\;\text{sin}\;\theta +2(f+d)}.$$ (18)

As the length of segment *EG* is |*EG*| = *l* sin *θ* tan *β*, the following equation can be derived:

$$\frac{\left|ab\right|}{\left|bc\right|}=\frac{\left|AF\right|}{\left|FG\right|}=\frac{l\;\text{cos}\;\theta +\left|EF\right|}{\left|EG\right|-\left|EF\right|}=1+\frac{l}{f+d}\;\text{sin}\;\theta =1+\frac{\left|O_{2}O_{3}\right|}{\left(\left|OO_{1}\right|+\left|O_{1}O_{2}\right|\right)}.$$ (19)

Then, the deviation ratio is given by

$$\tau =\frac{l}{f+d}\text{sin}\;\theta =\frac{\left|O_{2}O_{3}\right|}{\left(\left|OO_{1}\right|+\left|O_{1}O_{2}\right|\right)}.$$ (20)

Given the width and location of the line, the derivation ratio is proportional to *θ*. Moreover, lines with the same *z*-coordinate in the *CCS* have the same derivation ratio.

The width of line segment *ac*, which is the line profile on the image plane, can be derived as follows:

$$\frac{f}{f+d}=\frac{\left|ac\right|}{\left|AG\right|}=\frac{\left|ac\right|}{l\;\text{cos}\;\theta +l\;\text{sin}\;\theta \;\text{tan}\;\beta }\Rightarrow \left|ac\right|=\frac{fl}{f+d}(\text{cos}\;\theta +\;\text{sin}\;\theta \;\text{tan}\;\beta ).$$ (21)

The offset of the center is easily determined as

$$\Delta =\frac{fl^{2}}{{\left(f+d\right)}^{2}}(\text{sin}\;\theta \;\text{cos}\;\theta +{\text{sin}\;}^{2}\theta \;\text{tan}\;\beta ).$$ (22)

As *θ* and *β* are known, Eq (22) can be rewritten as

$$\Delta =\frac{\delta fl^{2}}{{\left(f+d\right)}^{2}},$$ (23)

where *δ* = (sin*θ*cos*θ*+sin^2^ *θ*tan*β*).

The partial derivatives of Δ with respect to *l*, *f*, and *d* are obtained as

$$\{\begin{array}{l} \Delta_{l}^{'}=\frac{2\delta fl}{{\left(f+d\right)}^{2}}\\ \Delta_{f}^{'}=\frac{\delta l^{2}(d^{2}-f^{2})}{{\left(f+d\right)}^{4}}\\ \Delta_{d}^{'}=\frac{-2\delta fl^{2}}{{\left(f+d\right)}^{3}} \end{array}.$$ (24)

The following properties can be deduced from Eq (24):

1. The offset is proportional to *l* and inversely proportional to *d*. Further, it is proportional to the focal length *f* when *d* > *f*, and inversely proportional to *f* when *d* < *f*. In general, *d* is far larger than *f*; therefore, the assumption that the offset is proportional to *f* can be treated as a fact in practice.
2. The offset is a function of *θ* and *β* when *f*, *l*, and *d* are determined. The relationship is shown in Fig 11.

**Fig 11:**
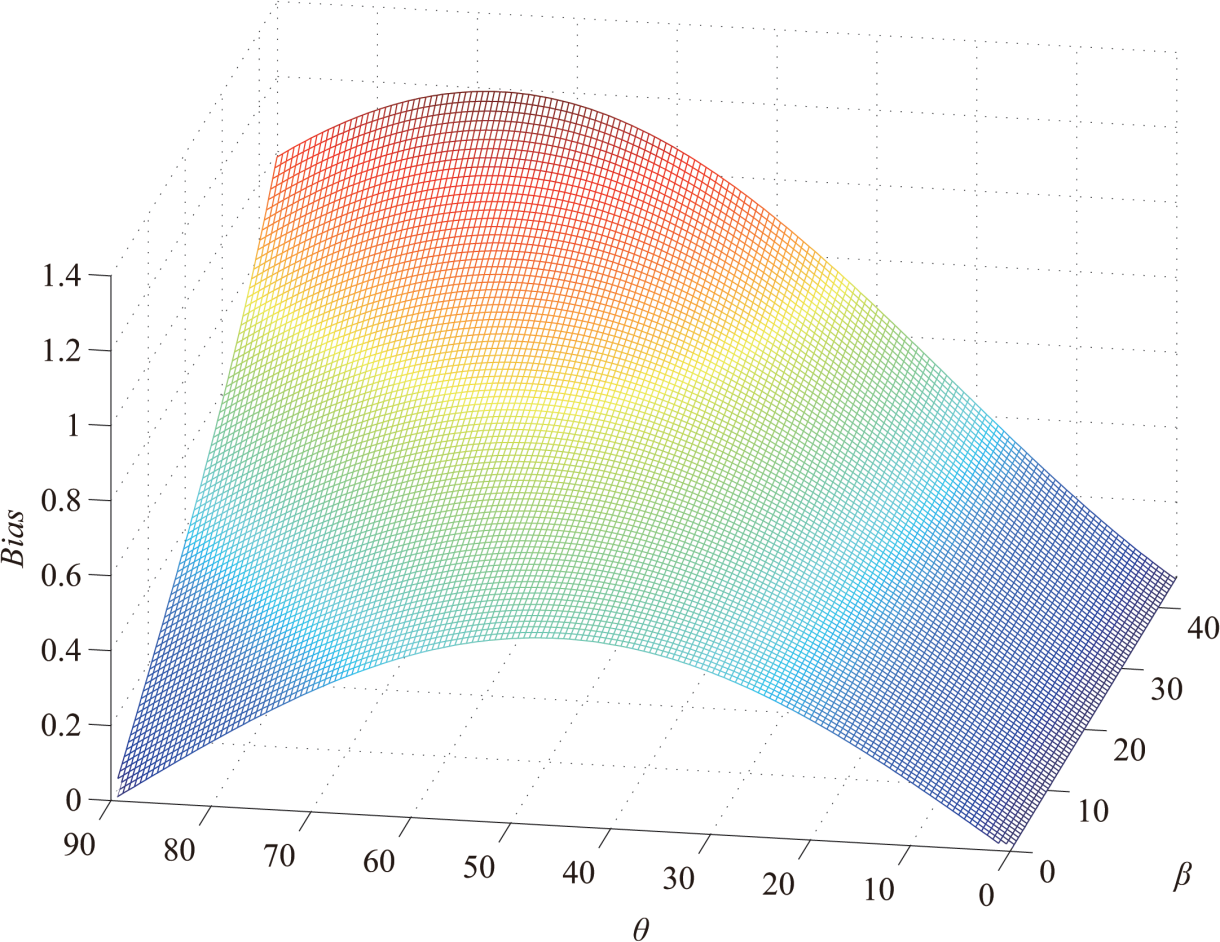
Perspective distortion as a function of *θ* and *β*.

Because we assume $\frac{fl^{2}}{{\left(f+d\right)}^{2}}$ to be 1 in Fig 11, the offset should be multiplied by a scale factor in practice. We can see that the offset is zero when *θ* is zero. Although the offset is proportional to *θ*, the available information will reduce as *l* decreases and *θ* increases. Synthesizing the related factors, *θ* should be minimized to obtain sufficient information and decrease the offset. If required, correction can be performed on the basis of Eq (22).

### B) Perspective distortion in measurement

In this section, we consider a typical vision measurement system, namely, the line-structured light vision system (*LSLVS*), as an example. In the measurement process of *LSLVS*, a light stripe is projected onto the target surface. The light stripe, which represents the available information, is captured for reconstruction.

The schematic of *LSLVS* is shown in Fig 12. The profile of the laser beam, i.e., the line segment *ab* in Fig 12, can be represented as the normal Gaussian line profile. The profile of the projection on the image plane is *AB*. Line segment *BC* is parallel to line segment *ab*, and it also satisfies the normal Gaussian distribution. Point *D* is the midpoint of *BC*, while line *DF* is parallel to line *AC* and intersects with line *AB* at point *F*. Because the projective angle of the laser projector (angle *Φ* in Fig 12) is small (in general, around 0.02°), the line segment *AB* can also be treated as the normal Gaussian line profile. Similarly, the midpoint of line segment *AB* can be treated as the projection (point *E*) of the center of the laser projector, i.e., point *E* and point *F* are approximately coincident.

**Fig 12:**
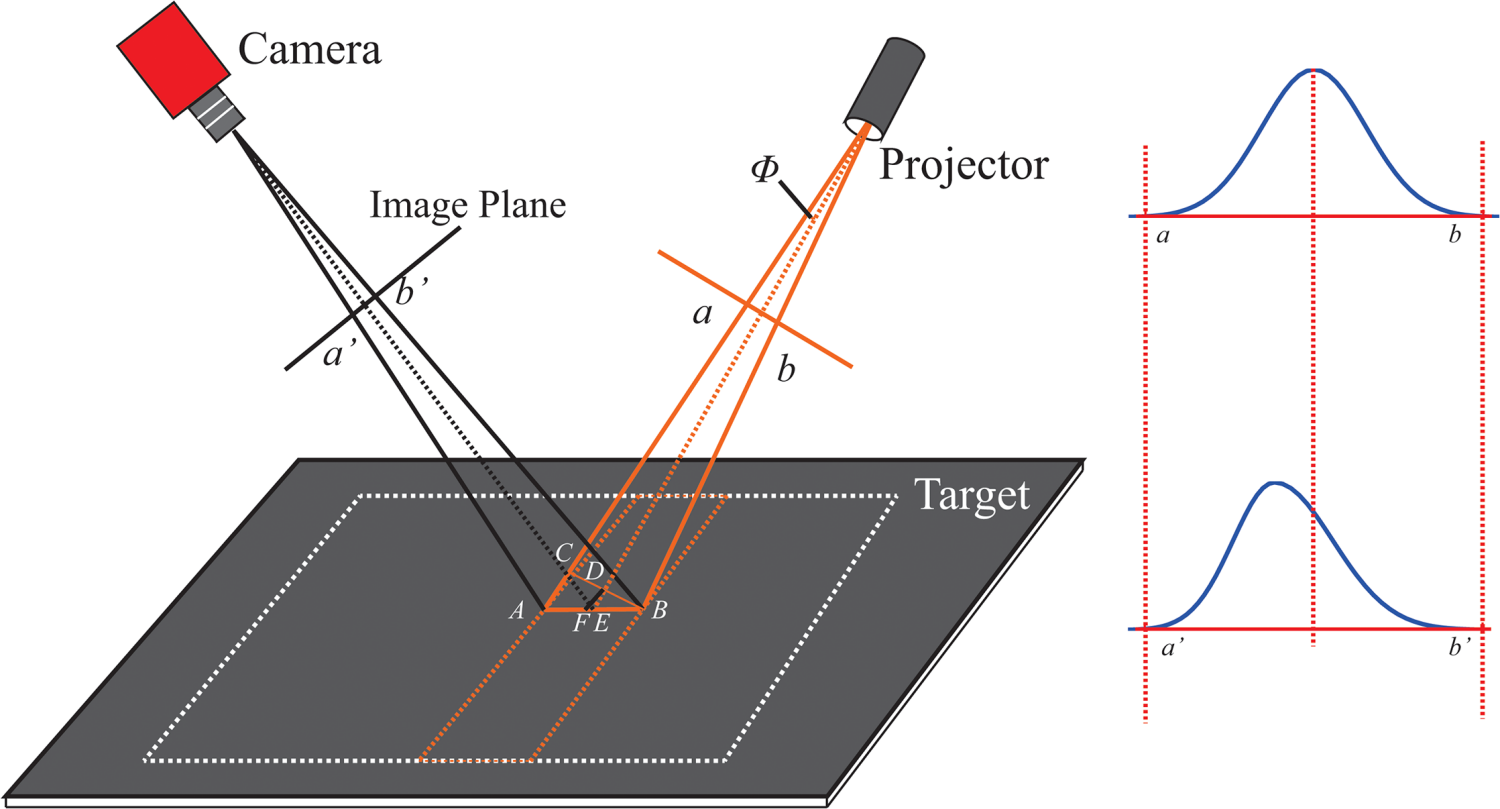
Schematic of line-structured light vision system.

Point *a’* and point *b’* are the projective points of point *A* and point *B* on the image plane. Line segment *a’b’* is not a Gaussian line profile, but it should be multiplied by a function of the Gaussian line profile. Then, the description of line segment *a’b’* is given by

$$f(x)=\{\begin{array}{l} e^{-\frac{F^{2}x^{2}}{2w^{2}}},(-\infty ,\text{sin}\;\theta *F]\\ a+(1-a)e^{-\frac{F^{2}x^{2}}{2w^{2}}},(\text{sin}\;\theta *F,+\infty ) \end{array}.$$ (25)

Owing to offset of the light stripe in the *x*-direction, Eq (25) is simplified as

$$f(x)=e^{-\frac{F^{2}x^{2}}{2w^{2}}},$$ (26)

where *F* = cos*θ* + sin*θ*tan*β*, and *θ* and *β* are defined as in Fig 10. For a vision measurement system, the normal *FOV* is less than 90° (e.g., a 1/3” *CCD* camera with a 2.8-mm lens); similarly, *β* is less than 45°. Then, Eq (26) can be rewritten as

$$f(x)={\left(e^{-\frac{x^{2}}{2w^{2}}}\right)}^{F^{2}}.$$ (27)

*F* and its gradient as a function of *θ* and *β* are shown in Fig 13.

**Fig 13:**
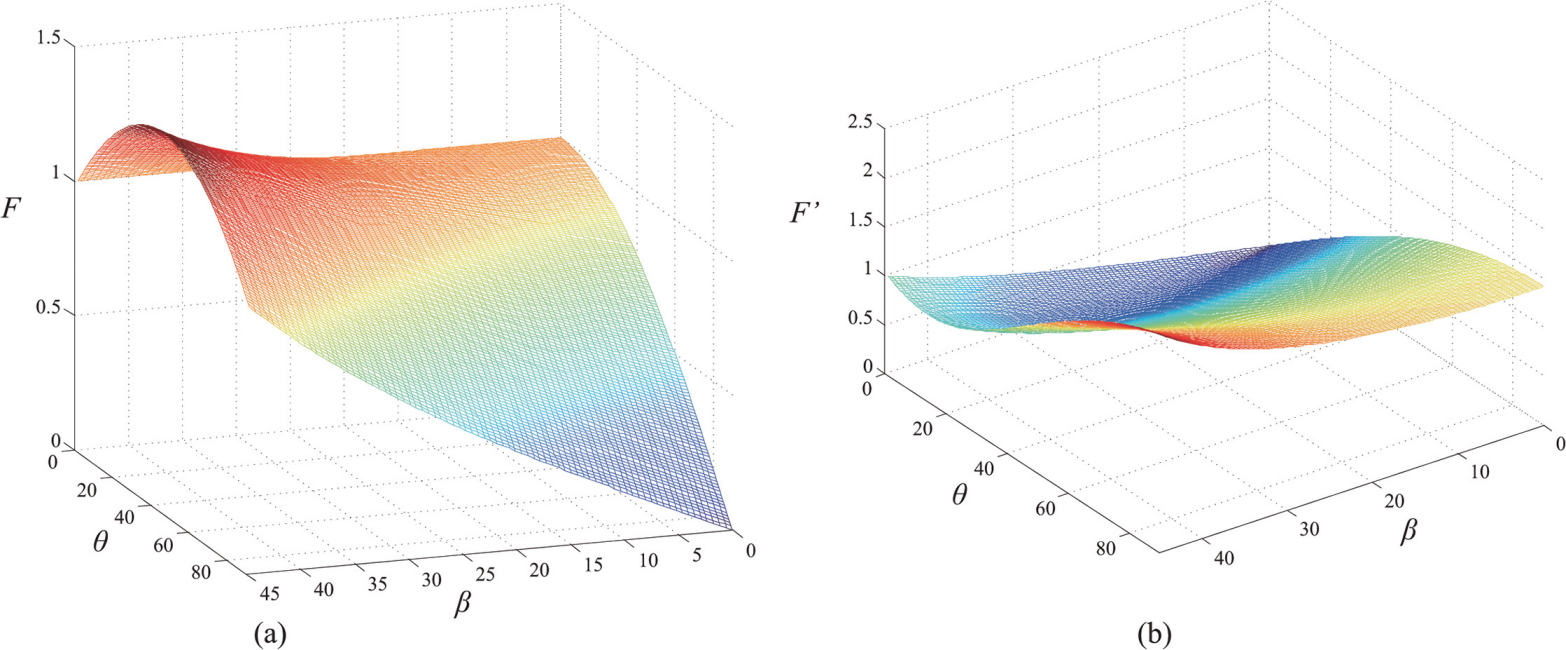
(a) *F* as a function of *θ* and *β*; (b) Gradient of *F* as a function of *θ* and *β*.

In Fig 13(A), the value of *F* is less than 1.5. Moreover, the continuity of *F* with a small gradient can be deduced from Fig 13(B). As there exists only a small variation in *θ* and *β in* the *CCS*, the variation of *F* is small, i.e., the offset is small. In the measurement process of *LSLVS*, the width of the laser stripe is small, whereas the distance from the lens to the target (*d*) is much greater than the focal length (*f*). Therefore, we obtain the relationship $\frac{fl^{2}}{{\left(f+d\right)}^{2}}\ll 1$. In other words, the offset due to perspective distortion is small. The geometrical center of the line image can be considered as the representation of the center of the line in the scene. In some special fields that require high precision, the line position can be corrected, if required, on the basis of the method described in Section 4.

## Experiment

### A) Images using the over-exposure model

In this section, we explain how the over-exposure model is used to extract the line position from an over-exposed image. In the field of medical image analysis, owing to over-exposure, the image of the line is always saturated in the negative direction (Fig 14(A)). During laser measurement, over-exposure is inevitable; Fig 14(B) and 14(C) show some examples. The line position is extracted using the over-exposure method, and the related results are plotted in Fig 14.

**Fig 14:**
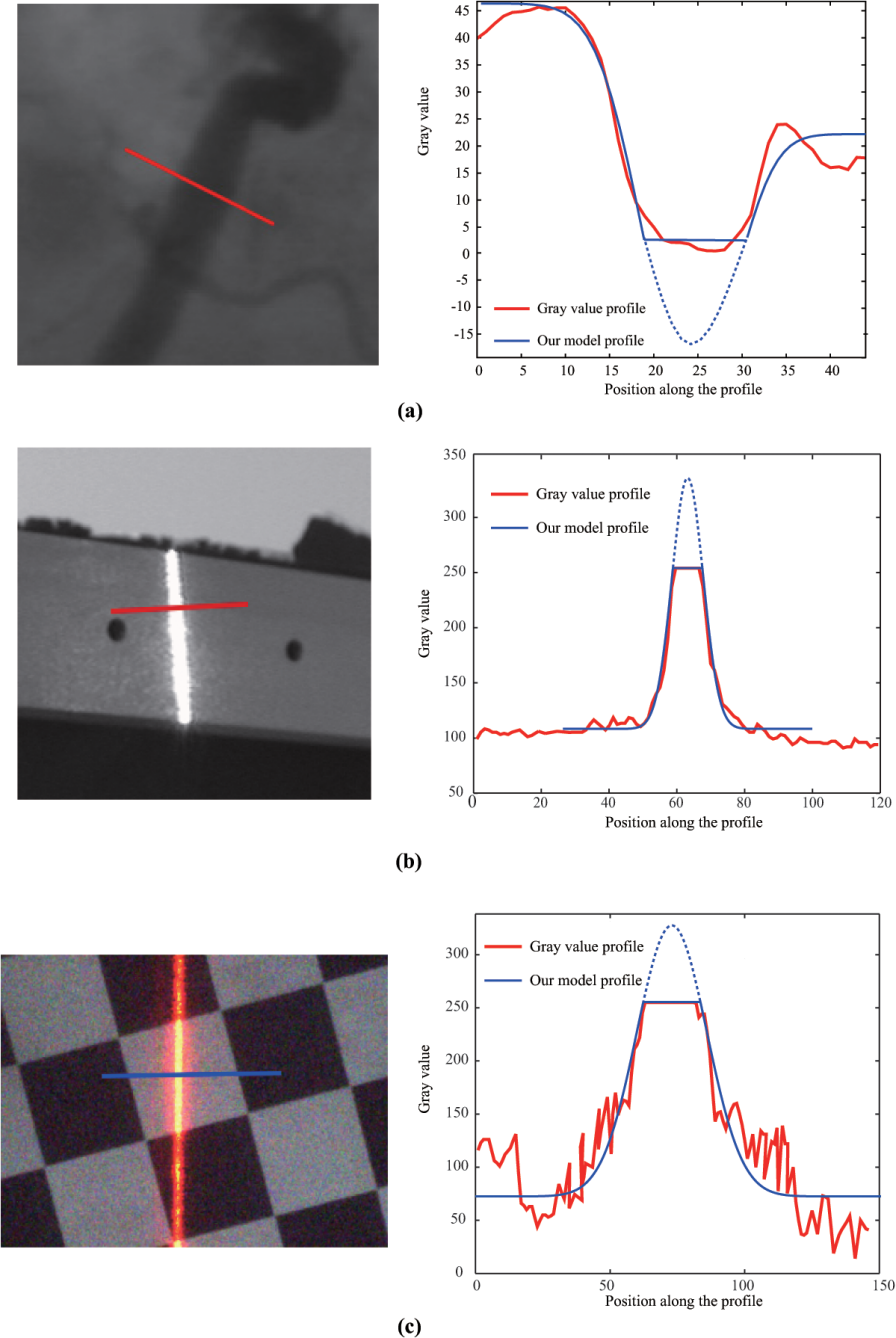
Application of the proposed method to (a) medical imaging (the image is taken from [27]); (b) image of a 1D target in the line-structured light vision system; (c) image of a planar target in the line-structured light vision system.

To verify the effect of exposure level on the proposed method, images were captured under different levels of exposure. Fig 15 shows the setup of the line-structured light vision system. In this system, *AVT F*-504*B* was employed as the camera for capturing images of the light stripe at different levels of exposure. The light stripe was projected onto the surface of an experimental workpiece. By controlling the exposure time, a series of images at different exposure levels can be captured. As the camera faces the surface of the experimental workpiece, the perspective distortion is negligible in this experiment. The results are shown in Fig 16.

**Fig 15:**
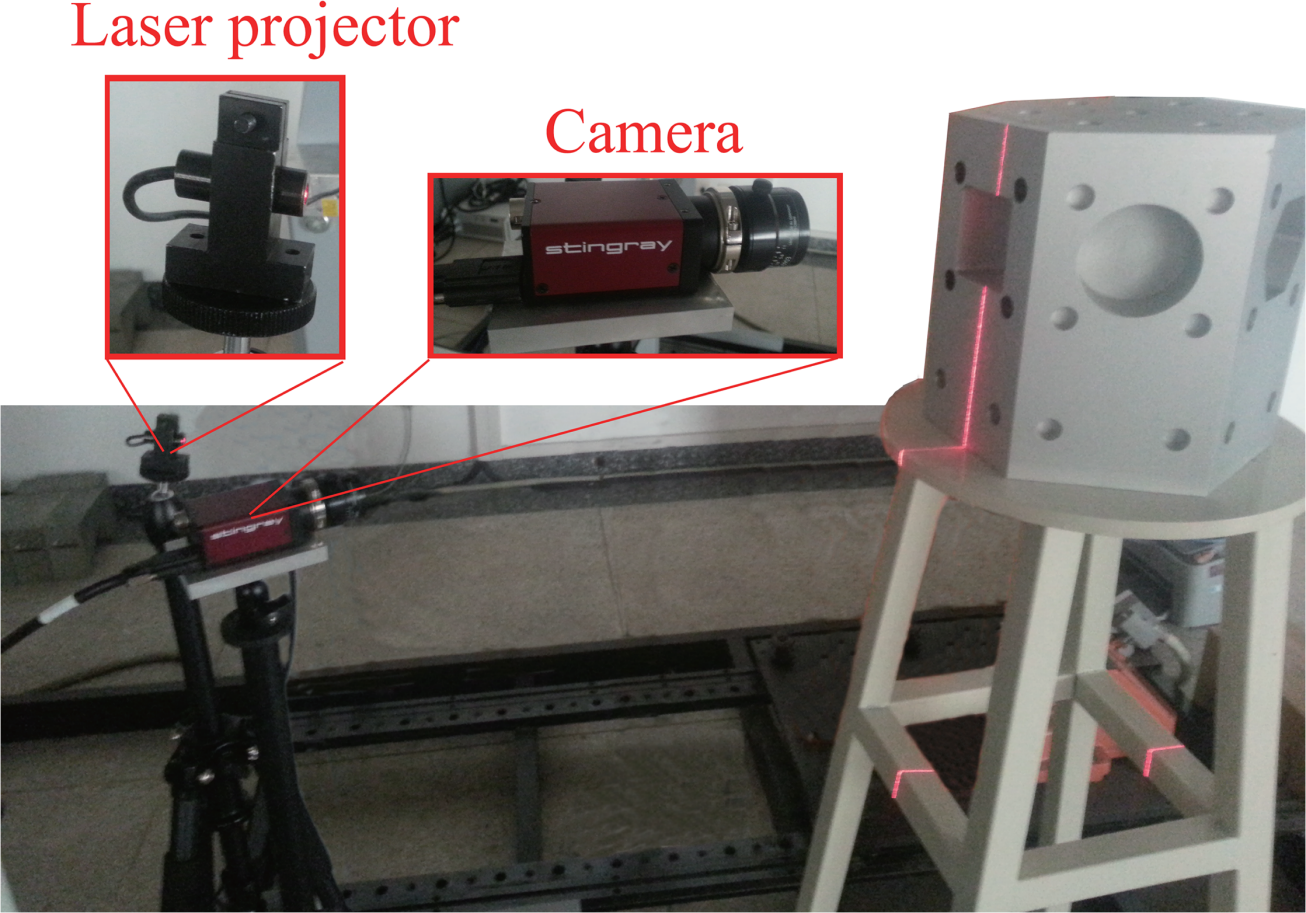
Structure of the over-exposure experiment.

**Fig 16:**
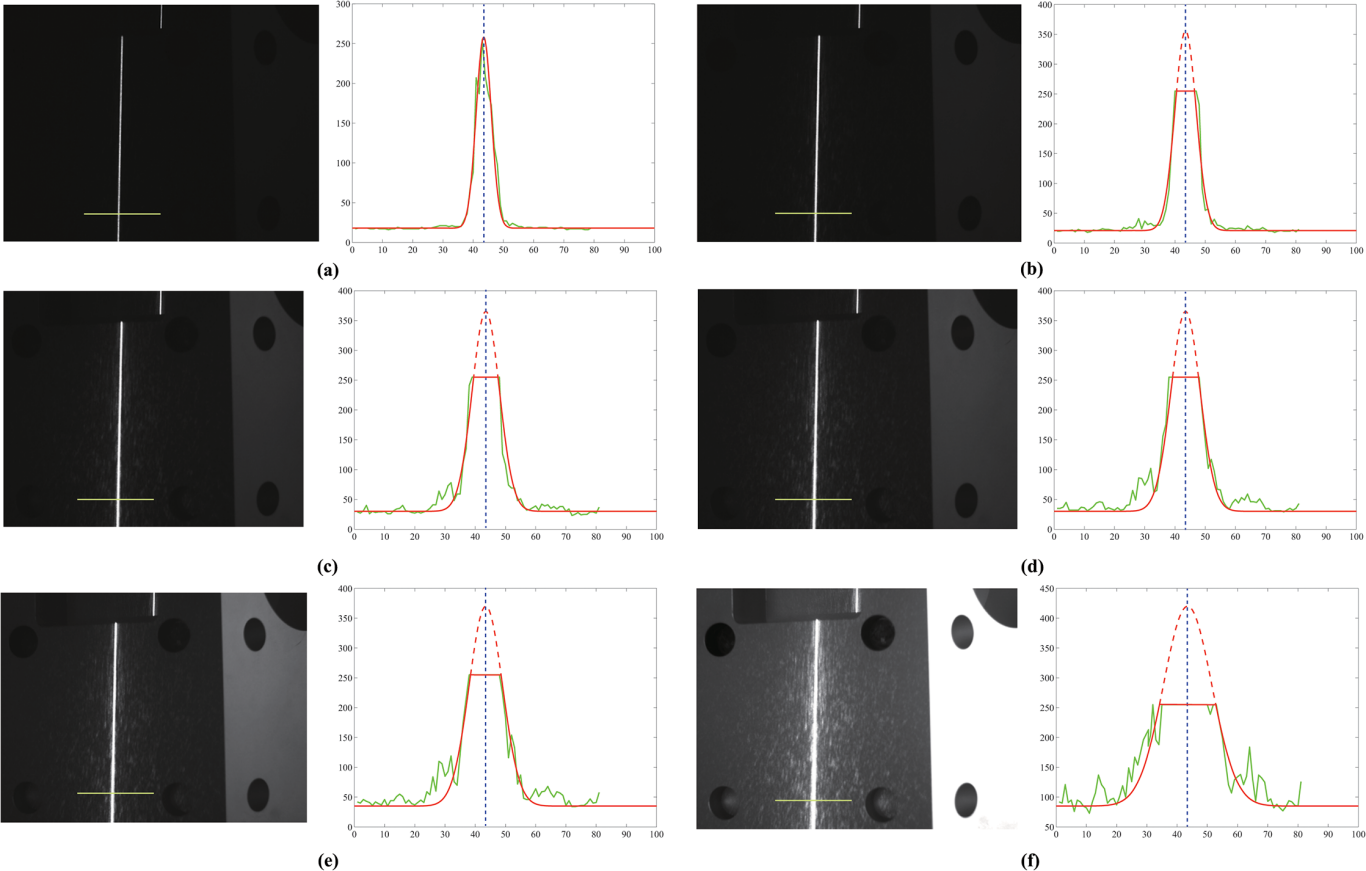
Extraction of the light stripe at different exposure levels. The green line represents the gray value of the profile, whereas the red line represents the fitting result. Extraction with exposure duration of (a) 70 ms; (b) 253 ms; (c) 590 ms; (d) 870 ms; (e) 1030 ms; (f) 2723 ms.

The images captured at different levels of exposure are shown in Fig 16. The exposure time of the image in Fig 16(A) is 70 ms, and the gray value of the line is unsaturated. Therefore, for Fig 16(A), extraction using the Gaussian model of Steger’s method is identical to that using the proposed method. Further, it is verified that the extraction accuracy is sufficiently high such that the obtained value can be regarded as the true value in this experiment. From Fig 16(A) to Fig 16(F), the exposure time increases, and the exposure becomes increasingly evident. Thus, the centers of these images (Fig 16(B)–16(F)) can be detected by the proposed method. Unlike the method described in [27], the proposed method detects the centers according to the fitting edges of the profile, and the result obtained is compared with the true value.

In order to compare with the detected result of other methods, the extractions were also performed using Steger’s method and the method mentioned in [23] (in Fig 16, the extractions using other methods are not plotted for the sake of clarity). The line centers using three different methods as a function of exposure time are listed in Table 1.

**Table 1:** Extraction results under various exposure times by three methods.

No.	Time of exposure (ms)	Gaussian line profile (pixel)		Method in [23] (pixel)		Over-exposure model (pixel)	
	———	Center	Error	Center	Error	Center	Error
	70	43.40	———	43.40	———	43.40	———
1	253	44.03	0.63	42.87	-0.53	43.17	-0.23
2	590	42.56	-0.85	41.98	-0.89	43.53	0.13
3	870	42.52	-0.88	41.06	-0.92	44.03	0.63
4	1030	43.10	-0.30	40.05	-1.01	44.24	0.84
5	2723	42.04	-1.36	38.78	-1.27	43.44	0.04
RMS	———	———	0.88	———	0.95	———	0.48

According to Table 1, the root mean square (RMS) error of the centers extracted by the proposed method is less than 0.5 pixels, while the RMS error of the centers extracted by the other two methods is less than 1.0 pixel. Although Steger’s method and the method in [23] also provide good results, they are less accurate than the over-exposure model. The corrected result is more precise than the uncorrected result by around 45.5%. Thus, the proposed method is capable of precisely detecting the center from an over-exposed image.

### B) Correction of perspective distortion

Because it is difficult to determine the center of the line, a planar target with zebra stripes is employed in our experiment. The image is captured at an angle of around 60°. In the experiment, the adjacent black and white stripes are treated as a group. In this case, the center is obvious because of the distinct contrast between black and white. Then, the group is processed as a single color (Fig 17(A)), and the image center of the group can be determined, while the corrected center can also be determined using Eq (20). The related result is shown in Fig 17.

**Fig 17:**
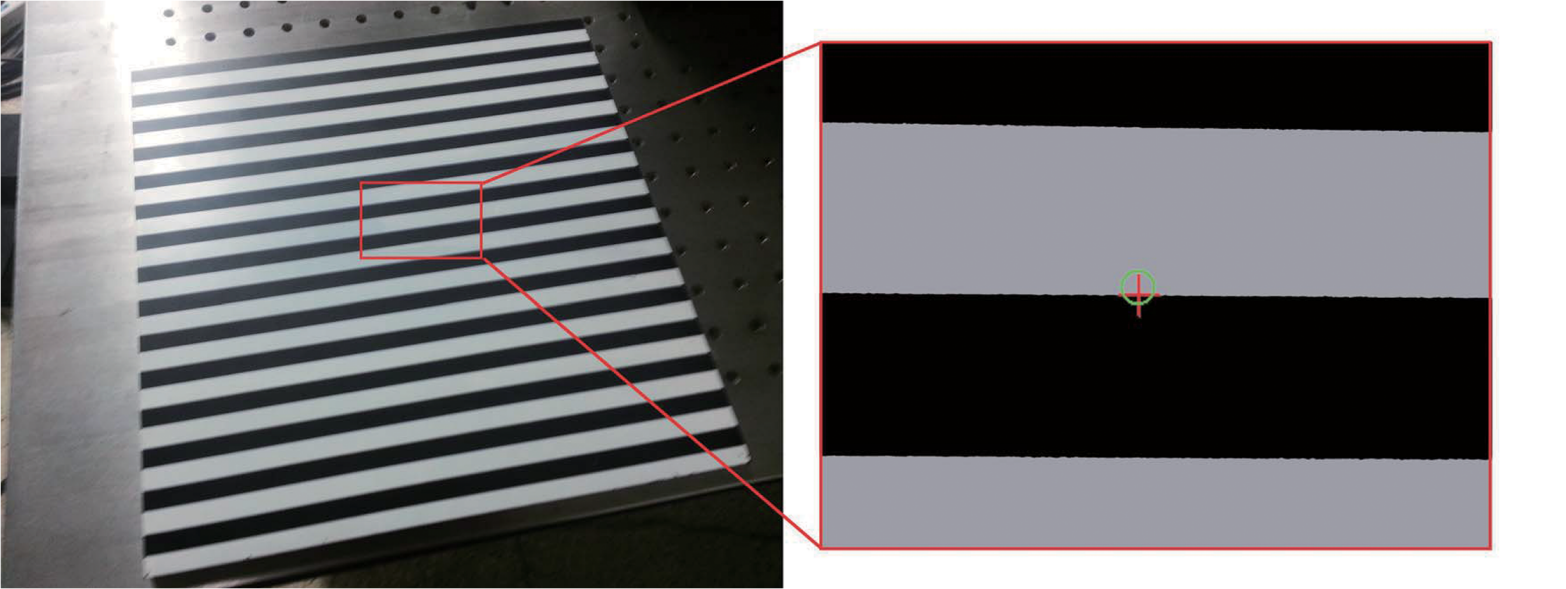
Effect of perspective distortion on extraction. (a) Processed image, (b) Original image. The green circle denotes the image center, whereas the red cross denotes the corrected one.

Furthermore, a series of experiments were conducted to evaluate the effect of perspective distortion on the extraction. Because it is difficult to measure the shooting angle, we merely increased the angle to observe the perspective distortion. We obtained the center of the line using the extraction method and then corrected based on the related properties. The ideal center is obtained as descripted above. As the extraction method descripted in our manuscript, the center of the line at one position can be obtained from its one-dimensional line profile. In this case, the center is simplified to one-dimensional space and the results are listed in Table 2.

**Table 2:** Extraction results and its corrected results.

No.	True Value (pixel)	Uncorrected (pixel)		Corrected (pixel)	
	————	Center	Error	Center	Error
1	52.18	52.40	0.22	52.38	0.20
2	52.18	52.64	0.46	52.36	0.18
3	52.18	52.79	0.61	52.37	0.19
4	52.18	53.15	0.97	52.40	0.22
5	52.18	53.33	1.15	52.43	0.25
6	52.18	53.54	1.36	52.35	0.17
7	52.18	54.00	1.82	52.28	0.10
8	52.18	54.38	2.20	52.45	0.27
9	52.18	54.59	2.41	52.48	0.30
RMS	———-	———-	1.44	———-	0.22

In general, the image center does not correspond with the projection of the scene center on the image plane. According to Table 2, the extraction is improved evidently after correction of perspective distortion. The root mean square (RMS) error of the extracted center after correction is about 0.22 pixels, while the RMS error of uncorrected extraction is about 1.44 pixels. In our experiments, the shooting angle increases from No.1 to No.9 gradually. In order to show evidently, the perspective distortion as a function of the shooting angle is shown in Fig 18.

**Fig 18:**
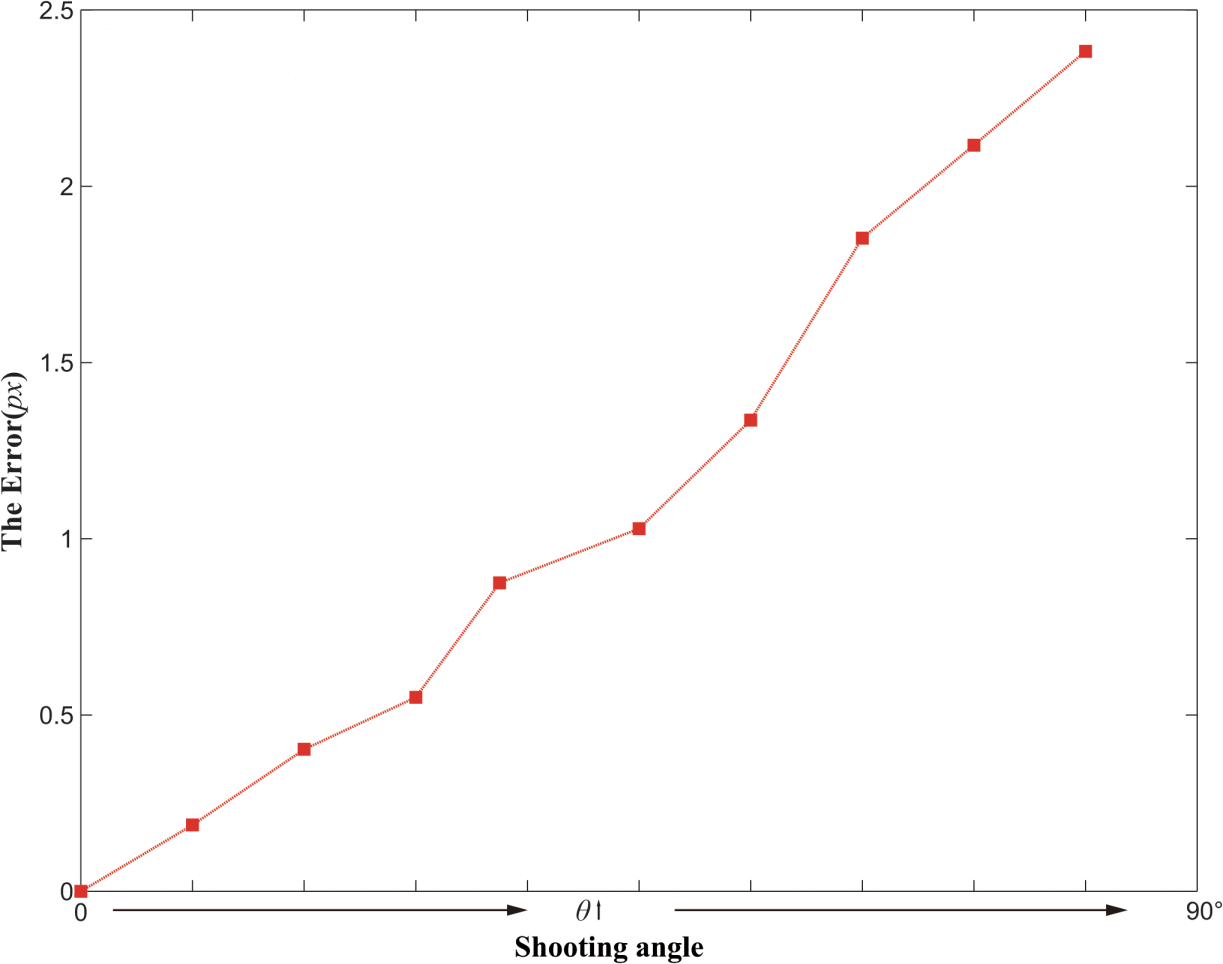
Perspective distortion as a function of the shooting angle.

It can be seen that, the perspective distortion cannot be neglected, especially when the shooting angle is large. After correcting based on Eq (20), the distortion is nearly eliminated. In practice, the correction should be performed according to specific requirements.

## Conclusion

In this paper, we proposed a method for correcting line positions extracted from an over-exposed image. Based on the Gaussian line profile of Steger’s method, a new line model was developed by incorporating over-exposure features. Accordingly, the proposed model was used to determine the line position from an over-exposed image. Simulations and experiments showed that the proposed model is more suitable than Steger’s method for line extraction from an over-exposed image in terms of its accuracy and its ability to detect the actual line center in the over-exposed image. We also analyzed the perspective distortion, which is inevitable during line extraction owing to the projective camera model employed in vision measurement. The perspective distortion can be rectified on the basis of the bias introduced as a function of related parameters. In addition, we discussed the properties of the proposed model and its application to vision measurement. In practice, a suitable model should be selected to correct line extraction, and the perspective distortion can be corrected according to specific requirements. Moreover, the proposed model can be used not only in vision measurement but also in other fields such as remote sensing and medical image analysis, where over-exposed images are frequently encountered.

Although the proposed line model is based on the Gaussian line profile, higher accuracy can be achieved by replacing the Gaussian line profile by a better extraction method. In addition, when the over-exposure is severe, the saturation value is small. In such cases, the proposed line model becomes ineffective, and it should be improved to obtain the actual line position. We plan to investigate such scenarios in future studies.
